# Huge thyroid adenoma with tracheomalacia

**DOI:** 10.1002/ccr3.1649

**Published:** 2018-06-17

**Authors:** Shan Jin, Wuyuntu Bao

**Affiliations:** ^1^ Department of General Surgery Affiliated Hospital of Inner Mongolia Medical University Hohhot Inner Mongolia Autonomous Region China

**Keywords:** huge thyroid adenoma, tracheal suspension technique, tracheomalacia

## Abstract

This case shows the importance of the early diagnosis of the tracheomalacia and the treatment of asphyxia in thyroid huge mass.

Huge thyroid adenoma is an extremely rare thyroid tumor. Thyroid lobectomy and isthmusectomy are the definitive treatment for the patients with a benign follicular adenoma. The tracheomalacia was found in operation, and we successfully performed the tracheal suspension technique. We recommend that preventing asphyxia is the prerequisite for operation success.

A 64‐year‐old man presented with a neck mass that had progressed in size for 30 years. In recently 3 months, presentation of dyspnea and dysphagia developed. There were no hoarseness and cough after drinking water. The physical examination revealed a huge, palpable, firm, and nontender mass in the neck, causing the tracheal displaced (Figure 1). Computed tomography (CT) showed a homogeneous large mass in the right thyroid, causing compression of the trachea and the esophagus, and causing the tracheal stenosis, diagnosed with thyroid adenoma (Figure 2). The patient was performed general anesthesia and intubated with difficulty. Complete surgical resection of the mass (right lobectomy and isthmusectomy) was performed (Figure 3). However, we found the tracheal cartilage collapses the condition in operation, and then, the tracheomalacia was confirmed via released the gas of tracheal tube pilot balloon. We successfully performed the tracheal suspension technique (Figure 4). Figure 4 suggested that there was still be found the trachea partly collapses between two tracheal cartilages after hanging the suspension sutures (purple line). Histopathological analysis revealed thyroid follicular adenoma with cystic medial necrosis and calcification (Figure 5). The patient recovered from dyspnea and dysphagia symptoms after surgery and discharged. Tracheomalacia is a condition characterized by excessive expiratory collapse due to the atrophy and/or reduction in tracheal elastic fibers of the tracheal wall or a reduction in the integrity of tracheal cartilage. At present, bronchoscopic visualization of dynamic tracheal or bronchial collapse remains “gold standard” for diagnosing tracheomalacia. Before surgery, we have not yet performed the bronchoscopic visualization, because CT not showed the tracheomalacia. Preventing asphyxia during operation and after surgery is important, for the tracheomalacia caused by thyroidectomy. Trachea suspension technique and tracheotomy are effective methods for saving asphyxiation during thyroidectomy. In this case, we are successfully saved the asphyxia, via performed the tracheal suspension technique.

**Figure 1:**
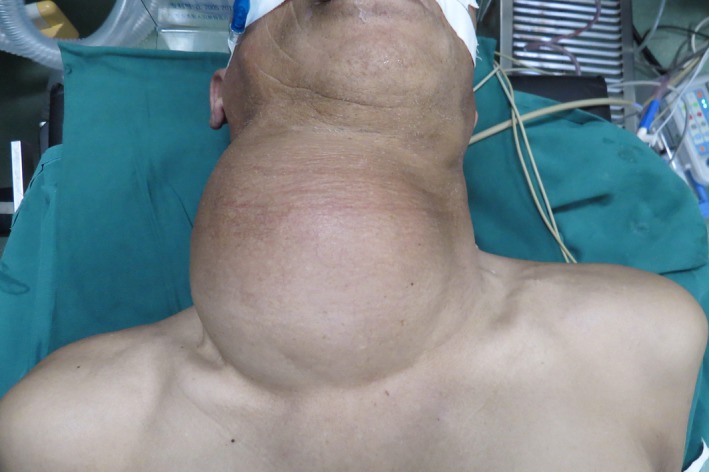
Clinical photograph of the neck huge mass

**Figure 2:**
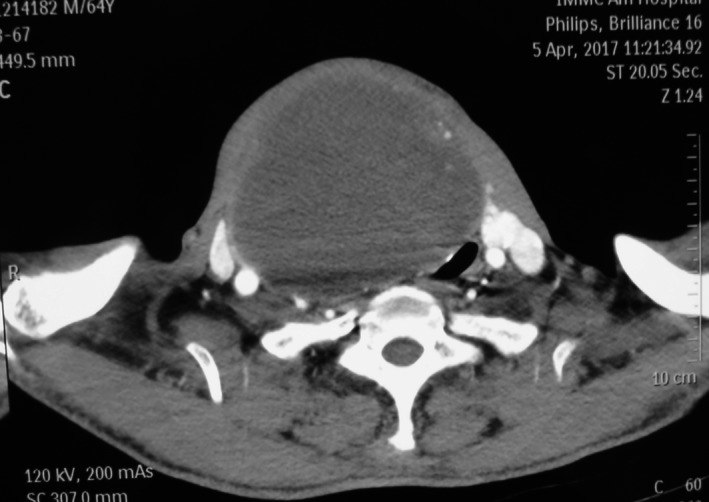
Computed tomography (CT) scan of the neck shows a homogeneous large mass in the right thyroid, causing compression of the trachea and the esophagus, and causing the tracheal stenosis

**Figure 3:**
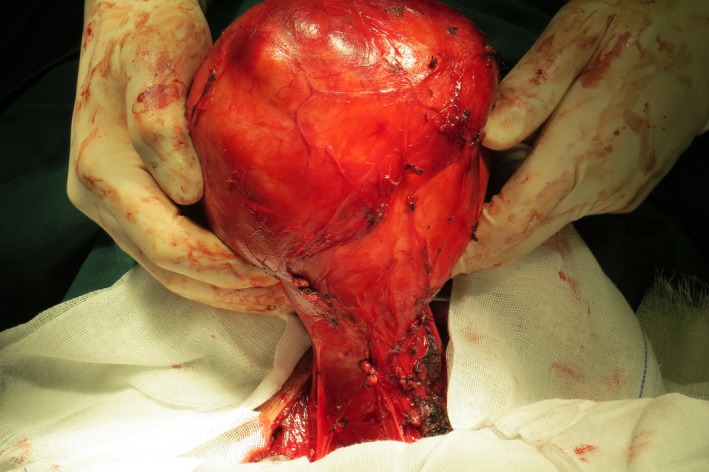
Image in operation

**Figure 4:**
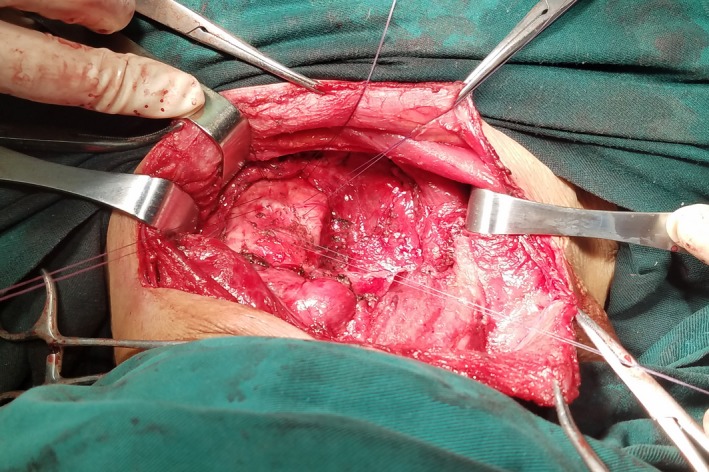
Tracheal suspension technique (purple line: suspension sutures)

**Figure 5:**
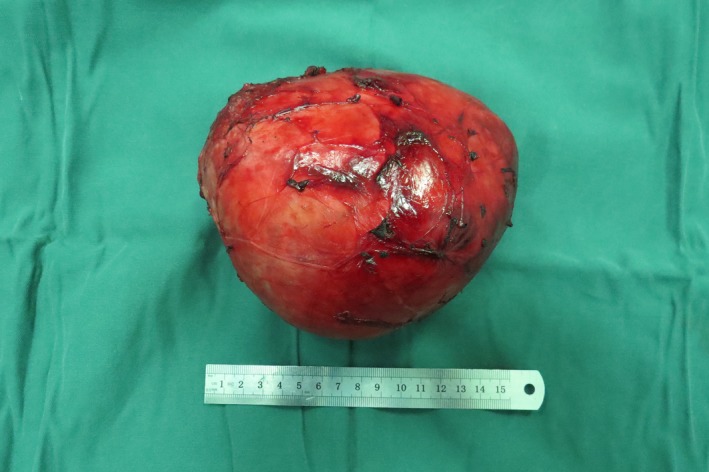
Huge thyroid adenoma

## CONFLICT OF INTEREST

None declared.

## AUTHORSHIP

JS and BW: contributed to conception and design, or acquisition of data, or analysis and interpretation of data.

